# Urinary proteomic pattern in female stress urinary incontinence: a pilot study

**DOI:** 10.1007/s00192-016-3033-5

**Published:** 2016-05-18

**Authors:** Marianne Koch, Goran Mitulovic, Engelbert Hanzal, Wolfgang Umek, Sonja Seyfert, Thomas Mohr, Heinz Koelbl, Rosa Maria Laterza

**Affiliations:** 1 Clinical Division of General Gynecology and Gynecological Oncology, Department of Obstetrics and Gynecology, Medical University of Vienna, Spitalgasse 23, 1090 Vienna, Austria; 2 Core Facility Proteomics, Clinical Institute of Laboratory Medicine, Medical University of Vienna, Spitalgasse 23, 1090 Vienna, Austria; 3 Science Consult Thomas Mohr KG, Guntramsdorf, Austria

**Keywords:** Leucine- rich alpha-2-glycoprotein, Lysosomal alpha-glucosidase, Plasma serine protease inhibitor, Uromodulin, TALPID3, Stress urinary incontinence, Urinary proteome

## Abstract

**Introduction and hypothesis:**

Previous studies aiming to identify specific pre-defined urine protein biomarkers for stress urinary incontinence (SUI) have not identified clinically important differences. The hypothesis of our study was that the global distribution of urinary proteins, the proteome, differs between women with and those without SUI.

**Methods:**

In this age-matched case–control study, we compared the urinary proteome of 20 women with SUI and 20 controls. Proteins were identified by applying high-performance liquid chromatography separation and tandem mass spectrometry detection. Data analysis was performed using Mascot 2.4.1 embedded in ProteinScape 3.1.

**Results:**

We identified 828 different proteins. The concentration of six of those showed a significant difference between urine samples of SUI patients and those of controls (*q* value < 0.25). Four proteins showed a higher abundance in SUI samples compared with controls: plasma serine protease inhibitor (logFC 1.11), leucine-rich alpha-2-glycoprotein (logFC 3.91), lysosomal alpha-glucosidase (logFC 1.24), and peptidyl-prolyl cis- trans isomerase A (logFC 1.96). We identified two proteins in lower abundance in SUI samples compared with controls: uromodulin (logFC −4.87) and TALPID3 (logFC −1.99).

**Conclusions:**

Overexpression of plasma serine protease inhibitor, leucine-rich alpha-2-glycoprotein, lysosomal alpha-glucosidase, and peptidyl-prolyl cis- trans isomerase A, and lower expression of uromodulin and TALPID3, in urine may be associated with female SUI.

**Electronic supplementary material:**

The online version of this article (doi:10.1007/s00192-016-3033-5) contains supplementary material, which is available to authorized users

## Introduction

Stress urinary incontinence (SUI) is defined as the complaint of “involuntary loss of urine on effort or physical exertion including sporting activities, or on sneezing or coughing” [1]. It has a widely varying estimated prevalence of 15–77 % of the female population. SUI represents both a psychological and an economic burden, and prevalence rates are expected to increase in the future, because of increasing life expectancy [2–5]. The classical epidemiology of SUI is well understood, with many environmental and life style risk factors identified, including age, obesity, parity, vaginal delivery, and family history [6–12]. Still, much of the etiology of SUI remains unclear, and it is difficult to predict which women are at risk.

Proteomics research offers one strategy to elucidate the etiology of SUI by identification of a significant and sufficient number of proteins, which provides the ability to avoid a pre-selection of candidate proteins. Many different serum, urine, and/or tissue protein markers have been investigated in the context of SUI. Almost all studies have targeted specific proteins as putative biomarkers, but with negative results. Previous studies have investigated the role of serum C-reactive protein [13], serum relaxin [14], and serum estradiol [15], without finding significant associations with symptoms.

To our knowledge, no study has yet investigated the complete urinary proteomic profile associated with SUI.

We aimed to determine a possible altered urinary protein profile in women suffering from SUI compared with healthy women, with the overall goal of providing new insights into the pathophysiology.

## Materials and methods

This was a prospective case–control study using proteomic analysis of urine samples from patients with SUI and healthy age-matched controls. Ethics approval was obtained from the ethics committee of the Medical University of Vienna (1788/2013). This trial was registered with ClinicalTrials.gov (NCT02023502).

The study was conducted between October 2013 and June 2015 as a cooperative project among the Department of Obstetrics and Gynecology, the Clinical Division of Urogynecology, and the Proteomics Core Facility, Medical University of Vienna. The inclusion criteria for stress incontinence cases were: a history of symptoms of SUI for at least 3 months (including a specific history of the complaint of involuntary leakage on effort or exertion or on sneezing or coughing), a positive provocation stress test (defined as an observed transurethral loss of urine simultaneous with a cough or Valsalva maneuver at a bladder volume of 300 ml), negative urine dipstick testing, age ≥ 18 years, patients capable of independent toileting, written informed consent, and at least one previous vaginal delivery. Exclusion criteria were: previous treatment for SUI (surgical or pharmacological), a history of overactive bladder symptoms and/or urinary incontinence other than SUI (tested using the ICIQ short form questionnaire) [16]; neurological disorders potentially affecting the urinary tract system, such as multiple sclerosis, Parkinson’s disease; pelvic organ prolapse stage ≥ II (International Continence Society classification), clinically significant bladder outlet obstruction and/or post void residual volume > 100 ml; a history of acute urinary retention or a history of repeated catheterizations, a history of bladder cancer or previous surgery to the urinary tract; acute or recurrent urinary tract infection and/or hematuria; a history of urinary tract stones; renal insufficiency and/or hepatic disease; a history of alcohol and/or drug abuse; pregnancy or lactation; and finally any patient with a serious medical condition. Participants in the control group met identical criteria, but with no symptomatic SUI (ICIQ short form score equal to zero), and a negative cough stress test. Urine samples were obtained once only without requirement for a specific time of day. Participants were given a sterile urine cup (maximum 50 ml) and asked to deliver the first-void urine. In addition, we retrieved blood samples from the peripheral veins of all participants to determine their creatinine, transaminase, and bilirubin status. Urine samples were stored in the refrigerator at 4 °C for a maximum of 1 h before being taken to the Clinical Institute of Laboratory Medicine (Proteomics Core Facility) for immediate processing.

Protein precipitation was performed by one of the authors according to the internally modified Wessel–Flüge method of protein precipitation and all solvents were kept at −20 °C. All working steps were performed on ice and centrifugation in a cooled centrifuge at +4 °C. Two ml of each urine sample were mixed with 6 ml methanol and 2 ml dichloromethane in a 50-ml Falcon tube and samples were vigorously vortexed. After adding 6 ml of water to each sample, solutions were vortexed again. Samples were subsequently stored at −20 °C for a minimum of 20 min for enhancement of protein precipitation. Phase separation was carried out by subsequent centrifugation for 5 min at 4,500 rounds per minute (rpm). The upper layer of the solution was then carefully discarded while keeping the interphase and lower layer, and an additional 6 ml of methanol were added before vigorous vortexing. Final centrifugation was performed for 5 min. The resulting supernatant was carefully removed and the remaining protein pellet was dried in the air. The dried protein pellet was later dissolved in 200 μl of 50 mM triethylammonium bicarbonate at pH 8.5 (TEAB). In cases where the protein pellet could not be properly dissolved in 200 μl of 50 mM TEAB, an additional 50–1,000 μl of 50 mM TEAB were added and the sample was sonicated by using the ultrasonic cell disruptor (Ultrasonic Cell Disruptor; Branson, Dietzenbach, Germany). Bradford protein assay was used to determine the protein concentration within the solution (mg/ml). The following protein digestion was performed as described by Mitulović et al. [17]. Briefly, disulfide bonds were reduced by the addition of dithiothreitol (DTT; 10 μg protein: 0.1 μg DTT) and incubation at 56 °C for 30 min. Alkylation of reduced proteins was performed by the addition of iodacetamide (IAA; 10 μg protein: 0.5 μg IAA) and incubation in darkness for 20 min at ambient temperatures. Upon reduction and alkylation, DTT was added to neutralize the excess of IAA and enable tryptic digestion, where sequence grade-modified trypsin (Promega, Mannheim, Germany; 1:10) was added to the sample, followed by overnight incubation at 37 °C. Tryptic digest was stopped by the addition of trifluoroacetic acid to a final concentration of 10 %. Upon tryptic digest, 30 μl of tryptic peptides were diluted with 20 μl of aqueous 0.1%TFA and 10 μl of sample were injected undiluted onto the chromatographic column. Peptide separations were performed using an Ultimate 3000 nano RSLC separation system (Thermo Fisher Scientific, Germering, Germany) coupled to the maXis Impact qToF mass spectrometer equipped with the captive spray electrospray source (Bruker, Bremen, Germany). The captive spray source was modified and a stainless steel needle (20 μm ID x 105 mm; Thermo Fisher Scientific, Vienna, Austria) was used instead of the default silica capillary for sample ionization, at 1.8 kV, and the source temperature was set to 150 °C. Samples were loaded onto a C18 trap column (300 μm ID x 5 mm Acclaim PepMap, 5-μm particle size, 100-Å pore size) using cooled (5 °C) aqueous 0.1%TFA at 30 μl/min as a loading mobile phase, and separated on a C18 nano separation column (75 μm ID x 250 mm Acclaim PepMap, 3-μm particle size, 100-Å pore size) operated at the flow rate of 300 nl/min. The gradient for nano-HPLC separation of tryptic peptides was generated using the following mobile phases: 5 % acetonitrile (AcN) in 0.1 % aqueous formic acid (FA) and 0.08 % FA in 50 % AcN, 30 % methanol, 10 % trifluoroethanol (TFE), 0.08 % FA.

All masses with a signal higher than 5,000 counts were submitted for fragmentation (MS/MS). MS/MS fragmentation was performed by applying a cycle time of 3 s, single charged peptides were excluded from fragmentation, and active exclusion was used for fragmented masses for 2 min. All MS/MS spectra were transformed into .mgf files for data analysis by applying an internal script from Data Analysis 4.1 (Bruker). Data analysis was performed using Mascot 2.4.1 (Matrix Science, London, UK) embedded in ProteinScape 3.1 (Bruker). Search parameters were as follows: the latest version of the IPI_human decoy database was used, trypsin was selected as the digestion enzyme, and two miscleavages were allowed. Monoisotopic peptide masses were searched with 50-ppm peptide mass tolerance and 0.5-Da fragment mass tolerance. The large mass tolerance for the database search was selected, although the actual mass accuracy of the data analyzed was <5 ppm. Carbamidomethyl on Cys was selected as a fixed modification and oxidation on Met as the variable peptide modification. Matches with Mascot scores >15, two peptides per protein, and a significance threshold of *p* < 0.05 were considered positive hits.

The sample size was planned as 20 patients per group (*n* = 40; false discovery rate 0.05, power 80 %, assumed proportion of true null hypotheses 0.95, and assumed standardized effect size 1; based on two-sample paired* t* test) and patients were matched for age (± 5 years). Similarities to tag count-based mRNA technologies led us to employ an overdispersed Poisson model combined with empirical Bayes methods, as commonly used to estimate mRNA tag abundance. Count data were loaded into R (version 3.1.3) and protein abundance was estimated by calculating peptide counts normalized to counts per million (cpm). Log2-fold change was estimated based on variance stabilized average log2 cpm values using the package edgeR. [18]. Resulting* p* values were corrected for multiple testing according to Bass [19]. A* q* value of <0.25 was considered statistically significant.

Statistical analysis of demographic data was computed applying independent* t* test for scaled variables (age, BMI, ICIQ score, number of vaginal deliveries) and Chi-squared test for nominal variables (status of menopause, presence of chronic disease).

The manuscript was structured according to the Strengthening the Reporting of Observational Studies in Epidemiology (STROBE) guidelines (for observational studies) and to the Minimum Information About A Proteomics Experiment (MIAPE) (for proteomic research).

## Results

One urine sample was retrieved from each participant (cases *n* = 20; controls *n* = 20) and included in the final analysis (total *n* = 40). Demographic data were similar in the case and control group (Table 1). We identified 6,415 proteins in the control group and 5,310 in the case group when identifications with one peptide per protein were included and identifications from all samples were taken into account. However, only proteins with at least two detected and identified peptides were selected for further statistical analysis. Thus, 828 individual proteins were entered into the statistical analysis.

**Table 1:** Demographic data of patients with stress urinary incontinence (SUI) versus controls

	Patients with SUI (*n* = 20)	Controls (*n* = 20)	*p* value
Age (years), mean (± SD)	49 (±9)	49 (±10)	0.961
BMI (kg/m^2^), mean (± SD)	28 (±6)	25 (±5)	0.119
ICIQ sum score, mean (± SD)	13 (±4)	0 (0)	0.000
Vaginal deliveries,* n* mean (± SD)	2.1 (±0.9)	1.9 (±0.7)	0.462
Chronic diseases,* n* (%)^a^	10/20 (50)	11/20 (55)	0.752
Menopause,* n* (%)			0.501
Premenopausal	15/20 (75)	12/20 (60)	
Postmenopausal	5/20 (25)	8/20 (40)	

*ICIQ-UI short form* International Consultation on Incontinence Modular Questionnaire

^a^Number of patients with chronic diseases (including hypertension, coronary heart disease, colitis, depression, gastritis, diabetes type II, glaucoma, chronic atrial fibrillation, asthma, Hashimoto thyroiditis, hyperthyroidism, tricuspidal valve insufficiency, factor V Leiden)

Six of the 828 proteins showed a significant difference in abundance in urine samples between SUI and controls (*q* value < 0.25; Table 2). Four proteins showed a higher abundance in SUI samples compared with controls: plasma serine protease inhibitor (logFC 1.11), leucine-rich alpha-2-glycoprotein (logFC 3.91), lysosomal alpha-glucosidase (logFC 1.24), and peptidyl-prolyl cis- trans isomerase A (logFC 1.96). Two proteins (gene symbol: UMOD; gene symbol: KIAA0586) presented a lower abundance in SUI samples compared with controls: uromodulin (logFC −4.87) and TALPID3 (logFC −1.99).

**Table 2:** Proteins with a significantly different abundance in the urine of patients with SUI compared with controls

Protein	Accession	Gene symbol	logFC	*q* value
Tax_Id=9606 Gene_Symbol=SERPINA5 plasma serine protease inhibitor	IPI00007221	SERPINA5	1.111	0.029
Tax_Id=9606 Gene_Symbol=LRG1 leucine-rich alpha-2-glycoprotein	IPI01012772	LRG1	3.909	0.019
Tax_Id=9606 Gene_Symbol=GAA lysosomal alpha-glucosidase	IPI00293088	GAA	1.237	0.062
Tax_Id = 9606 Gene_Symbol=UMOD uromodulin	IPI00640271	UMOD	−4.867	0.002
Tax_Id=9606 Gene_Symbol=PPIA peptidyl-prolyl cis-trans isomerase A	IPI00910407	PPIA	1.962	0.227
Tax_Id=9606 Gene_Symbol=KIAA0586 TALPID3	IPI01010584	KIAA0586	−1.992	0.227

*logFC* logarithm of expression 1 (cases) minus the logarithm of expression 2 (controls) to basis 2 (log2(Exp1)-log2(Exp2)=logFC)

## Discussion

This pilot study identified six putative SUI-specific urinary proteins, to our knowledge for the first time.

Plasma serine protease inhibitor (SERPINA5), leucine-rich alpha-2-glycoprotein (LRG1), lysosomal alpha-glucosidase (GAA) and peptidyl-prolyl cis-trans isomerase A (PPIA) were more highly expressed in urine samples from women with SUI. Plasma serine protease inhibitor (SERPINA5) is usually present in urine in very low concentrations and serves, among other functions, as a pro-inflammatory factor. Recent publications regarding diverse medical conditions, including pediatric leukemia, breast cancer, HIV infection, and hepatocellular carcinoma have identified the role of SERPINA5 [20–23]. Leucine-rich alpha-2-glycoprotein, a secreted protein normally present in plasma, is involved in nonspecific inflammatory and cancer processes. It has recently been described in the context of ulcerative colitis activity, invasive bladder cancer, biliary tract cancer, lung cancer, pancreatic cancer, heart failure, neutrophilic granulocyte differentiation, and autoimmune diseases [24].

Lysosomal alpha-glucosidase (GAA) is a protein essential for the degradation of glycogen to glucose in lysosomes, which is present in all tissue cell types. Mutations in the respective gene result in Pompe disease, a condition in which a lack of lysosomal alpha-glucosidase leads to the intralysosomal accumulation of glycogen, which consequently disables heart and skeletal muscles. The protein has also been identified as a potential biomarker for gut wall integrity in infants with necrotizing enterocolitis, an inflammatory process involving intestinal tissue [25].

Peptidyl-prolyl cis-trans isomerase A is involved in inflammatory processes/immunomodulation and induction of interleukin-6 release from macrophages. Recent publications have discussed an involvement in type II diabetes mellitus, vascular disease, and gastric adenocarcinoma [26].

The proteins uromodulin and TALPID3 had lower expression in SUI samples. Among other functions, uromodulin is involved in the prevention of urinary tract infection, water/electrolyte balance, and kidney innate immunity, and is usually highly abundant in the urine of healthy humans [27]. TALPID3 is required for ciliogenesis and sonic hedgehog/SHH signaling [28].

We do not imply to have found biomarkers for SUI, but rather a group of proteins with a significantly different abundance in SUI patients compared with controls. By investigating the known functions, tissue specificities and interactions of the specific proteins, we may be able to gain insight into the potential mechanisms of the pathophysiology and etiology of SUI. Proteins that were identified with significantly higher abundance in SUI samples, are described as being involved in inflammatory processes and cancer development, whereas those with a significantly lower abundance usually seem to have a protective effect in the urinary tract system.

The strengths of this study include that urine samples were retrieved from a population with very strict inclusion and exclusion criteria to avoid confounding factors. Demographic data showed that there was no significant difference between the case and control group regarding BMI, age, and parity (Table 1). Urine samples were processed according to a standardized protocol within a very short time frame after collection (maximum 1 h). Moreover, all steps of sample processing were consecutively executed by two of the authors, which reduces a potential inter-observer bias and provides consistency in sample handling. Whereas previous studies on SUI have tested serum and/or urinary proteins as potential biomarkers for SUI, there has been no other study, to our knowledge, that has aimed to compare the complete urinary proteome of patients with SUI with that of healthy controls. Preceding studies have targeted specific proteins suspected to play a role in the development of SUI (e.g., serum C-reactive protein, relaxin, estrogen, etc.) [14, 15].

By applying liquid chromatography and mass spectrometry, we were able to possibly identify a significant and sufficient number of proteins present in one urine sample, which provides the ability to avoid a pre-selection of candidate proteins. The main limitation of this study is that we only retrieved and investigated one urine sample per patient. Therefore, we cannot avoid the bias that the urinary proteome potentially changes in one individual dependent on, for example, daytime, food intake, method of retrieval, etc. However, there is no guideline for urine sample collection regarding subsequent proteomic analysis and little is known about circadian urinary proteome changes or external influences.

Another limitation is the incomplete “humane proteome mapping.” Despite the efforts of the research community to identify and characterize all human proteins, this project has not yet been completed. Therefore, it is possible to identify proteins that have not yet been characterized, but which are hypothesized as products of specific genes owing to the similarity of their peptide chains to known proteins.

Last, by investigating the urinary proteome only at one time point, we cannot draw a conclusion on whether the significantly differently expressed proteins are a consequence of the pathological process, or whether they themselves are directly involved in causal processes.

The relevance of these results regarding the pathogenesis of SUI, focusing on protein interactions, needs to be more broadly investigated and the results of this pilot study need to be replicated in a different population.

## Electronic supplementary material

Below is the link to the electronic supplementary material.

ESM 1 Data of 828 proteins, which entered statistical analysis. logFC (logFold Change), logCPM (variance stabilized log2 of counts per million), LR (Likelihood Ratio), PValue, FDR (False Discovery Rate), QValue, Accession, Protein (XLSX 150 kbData of 828 proteins, which entered statistical analysis. logFC (logFold Change), logCPM (variance stabilized log2 of counts per million), LR (Likelihood Ratio), PValue, FDR (False Discovery Rate), QValue, Accession, Protein (XLSX 150 kb)

ESM 2 List of proteins identified per urine sample. Accession, Protein, Molecular Weight (kDa), Mascot Score, Sequence Coverage (%) (XLS 2573 kb)
